# Patient‐ and caregiver‐reported barriers to chemotherapy in nine sub‐Saharan African countries: A cross‐sectional survey among population‐based registries

**DOI:** 10.1002/ijc.70309

**Published:** 2025-12-30

**Authors:** Tamara König, Nikolaus Christian Simon Mezger, Ole Stoeter, Phoebe Mary Amulen, Margaret Borok, Gladys C. Chesumbai, Moudiongui MBoungou Dimitry, Ima‐Obong Ekanem, Adugna Fekadu, Bakarou Kamaté, William Muller, Alex Alain Kabena Nzambikolo, Abidemi Omonisi, Furaha Serventi, Markus Wallwiener, Biying Liu, Donald Maxwell Parkin, Pablo Sandro Carvalho Santos, Eva Johanna Kantelhardt, Eric Sven Kroeber

**Affiliations:** ^1^ Global & Planetary Health Working Group, Center of Health Sciences Medical Faculty of the Martin Luther University Halle‐Wittenberg Halle (Saale) Germany; ^2^ Kampala Cancer Registry, Department of Pathology: College of Health Sciences Makerere University Kampala Uganda; ^3^ Zimbabwe National Cancer Registry College of Health Sciences, University of Zimbabwe Harare Zimbabwe; ^4^ Eldoret Cancer Registry Moi Teaching and Referral Hospital Eldoret Kenya; ^5^ Brazzaville Cancer Registry Brazzaville Congo; ^6^ Department of Pathology, Calabar Cancer Registry University of Calabar Teaching Hospital Calabar Nigeria; ^7^ Addis Ababa City Cancer Registry, Radiotherapy Center Tikur Anbessa Hospital, Addis Ababa University Ethiopia; ^8^ Bamako Cancer Registry Bamako Mali; ^9^ Mbeya Cancer Registry Mbeya Referral Hospital Mbeya Tanzania; ^10^ Institut de Cancérologie d'Akanda Registre du Cancer du Gabon Libreville Gabon; ^11^ Ekiti Cancer Registry, Department of Medicine Ekiti State University Teaching Hospital Ado‐Ekiti Nigeria; ^12^ Moshi Cancer Registry, Kilimanjaro Christian Medical Centre, Cancer Care Centre Moshi Tanzania; ^13^ Department of Gynaecology Martin Luther University Halle‐Wittenberg Halle (Saale) Germany; ^14^ The African Cancer Registry Network Oxford UK; ^15^ Nuffield Department of Population Health University of Oxford Oxford UK; ^16^ International Agency for Research on Cancer Lyon France

**Keywords:** ageing, barriers to care, cancer care, chemotherapy, population‐based registration, sub‐Saharan Africa

## Abstract

Chemotherapy is an essential component of cancer treatment, as outlined in the National Comprehensive Cancer Network (NCCN) guidelines for Sub‐Saharan Africa (SSA). Lack of access to treatment is a key driver of impaired survival rates. This study assessed patient‐perceived barriers to chemotherapy in SSA according to the five dimensions of access to care: availability, accessibility, accommodation, affordability, and acceptability. Telephone interviews were conducted with 553 randomly selected cancer patients (or caretakers), registered between 2018 and 2019 in 11 urban population‐based cancer registries across nine countries in SSA. Malignancy types included breast, cervical, prostate, and colorectal cancer; non‐Hodgkin lymphoma; and Kaposi sarcoma. Patients rated barriers using a 3‐point Likert scale. Barriers to chemotherapy and their associations with patient characteristics were analysed using multivariate ordinal regression analysis. Major barriers included accessibility (cost of transport), affordability (cost of treatment, being absent from home), and acceptability (lack of knowledge/awareness and fear of treatment). Results varied between countries: affordability was especially severe in the Republic of Congo, while in Gabon, fear of treatment prevailed. Knowledge and awareness were particularly concerning in Ethiopia and Zimbabwe. A combined educational level and self‐reported wealth variable, and national human development index (HDI) were consistently associated with reported barriers. Overall, 58.6% of participants received chemotherapy, while 13.2% were recommended chemotherapy but did not receive it. A higher HDI correlated with an increased probability of receiving treatment. A complex set of barriers influenced patients' non‐receipt of treatment. Regionally adapted strategies, including psychosocial support, financial assistance for vulnerable groups, and education, are essential to improve treatment uptake in SSA.

## What's New?

Increasing cancer incidence and mortality in low‐ and middle‐income countries has heightened concerns about limited resources and barriers to care. This challenge is particularly urgent in Sub‐Saharan Africa (SSA), where cancer rates are rising sharply. Here, data from population‐based cancer registries in nine SSA countries was assessed to better understand patient‐reported barriers to chemotherapy access. Analyses identified financial constraints, fear, and limited knowledge and awareness as substantial barriers. Educational level, self‐reported wealth, and national human development index were consistently linked to reported barriers. The findings underscore the need to address socioeconomic inequalities and treatment‐related concerns to ensure fair chemotherapy access in SSA.

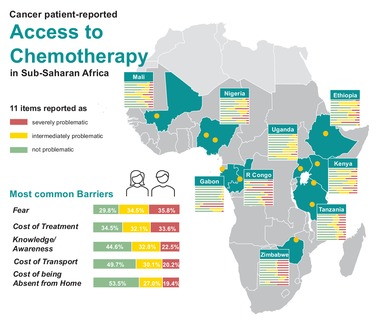

**Abbreviations**
AFCRN: African Cancer Registry Network
CI: confidence interval
HDI: human development index
HIC: high income countries
ICD: International Statistical Classification of Diseases and Related Health Problems
LMIC: low‐ and middle income countries
NCCN: National Comprehensive Cancer Network
NHL: non‐Hodgkin lymphoma
OR: odds ratio
SSA: Sub‐Saharan Africa

## 1 INTRODUCTION

Cancer incidence rates in Sub‐Saharan Africa (SSA) are projected to increase drastically, more than doubling between 2018 and 2040, partly due to demographic, reproductive, and lifestyle changes.^1^, ^2^ Although global cancer incidence rates are rising, mortality rates differ significantly between high‐income countries (HICs) and low‐ or middle‐income countries (LMICs).^3^ Studies on breast cancer indicate declining mortality rates in most HICs, while rates are increasing in LMICs.^4^

Besides early detection and diagnosis, timely access to cancer‐directed treatment is essential to improving outcomes for patients with cancer.^4^, ^5^ Treatment gaps in SSA are substantially larger than those in HICs.^6^, ^7^ These gaps are partly due to shortcomings in screening and early diagnosis, which result in delayed or missed treatment initiation.^2^, ^6^, ^7^, ^8^, ^9^ To streamline efforts in providing evidence‐based treatment to patients in LMICs, the NCCN has published harmonised oncological guidelines that take into account the limited availability of resources.^11^, ^12^ Nevertheless, many patients, even after obtaining a diagnosis, do not receive guideline‐concordant cancer‐directed treatment, which severely compromises survival rates.^13^, ^14^, ^15^, ^16^, ^17^

Research on barriers and enablers to treatment predominantly focuses on reasons for late diagnosis, such as lack of participation in screenings, and is often limited to breast or cervical cancer in individual countries.^5^, ^6^, ^8^, ^9^, ^18^, ^19^ In this study, we used a population‐based, multi‐country approach to assess self‐perceived barriers to chemotherapeutic treatment after diagnosis among adult patients with cancer living in SSA. We focused on the most common malignancies in the region: breast, cervical (uterine), prostate, and colorectal cancer; non‐Hodgkin lymphoma (NHL); and Kaposi sarcoma.

## 2 METHODS

### 2.1 Study population and data collection

We conducted a retrospective, cross‐sectional, multi‐centre survey, collecting data from 11 population‐based cancer registries across nine countries in SSA. These registries were located in the Central (Brazzaville, Republic of the Congo; Libreville, Gabon), Eastern (Addis Ababa, Ethiopia; Eldoret, Kenya; Kilimanjaro and Mbeya, Tanzania), Western (Bamako, Mali; Calabar and Ekiti, Nigeria), and Southern (Zimbabwe National Registry) regions. Due to travel restrictions during the COVID‐19 pandemic, the preparation, organisation, administration, and training of data collectors for this study were conducted via online video meetings. Data collection was performed by employees of the local cancer registries. All registries used the International Agency for Research on Cancer's CANREG software for epidemiological cancer registry management. Although most registries are based in urban areas, the registry in Zimbabwe covers the entire country. We included patients registered between January 2018 and December 2019 who were diagnosed with female breast cancer (ICD‐10: C50), cervical cancer (C53), prostate cancer (C61), colorectal cancer (C18–20), NHL (C82–85, C96), or Kaposi sarcoma (C46) (Data S1A).

### 2.2 Questionnaire design and target variables

The questionnaire was designed to capture the following aspects: sociodemographic characteristics of the patients, pre‐diagnosis history, diagnosis, chemotherapy treatment status (not recommended, recommended but not received, or received), perceived barriers to accessing treatment, and the patient's social environment.

The status of chemotherapy receipt was solely patient‐reported; verification by medical record review has not been possible due to the ongoing COVID‐19 restrictions during the time of data collection. Before participants were asked about barriers, we inquired whether they received surgery, radiotherapy, or hormone therapy. Data collectors were trained to support the participants in distinguishing between chemotherapy and hormone therapy (especially breast and prostate cancer). We included patients who reported not being recommended chemotherapy into the analysis due to the uncertainty of whether a non‐recommendation was medically justified or potentially influenced by non‐clinical factors (e.g., financial concerns by the physician). Furthermore, patients without a recommendation might have engaged in discourses about chemotherapy and developed opinions, which are relevant for understanding systemic and perceptual barriers to care.

Questions about barriers to treatment were grouped using the framework of the 5‐A model on access to care established by Penchansky and Thomas,^20^ which identifies *availability*, *accessibility*, *affordability*, *accommodation*, and *acceptability* as central aspects (Table 1). A 3‐point Likert scale (‘unproblematic’, ‘intermediate’, or ‘severely problematic’) was used to identify barriers to chemotherapy (Data S1E). In this study, the term *impeding* was used to refer to patients who selected either ‘intermediate’ or ‘severely problematic’. An interdisciplinary team of physicians and public health experts developed the questions in close collaboration with leaders of the participating cancer registries. The survey was piloted among 60 patients in Zimbabwe, and the questionnaire was adapted accordingly, with minor changes made to the structure, order of questions, and barriers included. When necessary, the questionnaire was translated from English or French into local languages.

**TABLE 1:** The five dimensions of access following the Access to care framework established by Penchansky and Thomas^18^.

Dimension	Definition	Items
Availability	The presence of a health facility offering chemotherapy treatment and that is therefore equipped to meet the needs of the patient.	Availability of chemotherapy facility
Accessibility	Patient's prospects to reach the geographical location of the health facility offering chemotherapy including the availability of resources (e.g., financial means and time to use public transport).	Availability of transport Cost of transport to health facility
Affordability	The financial abilities of a patient to afford treatment and associated costs (e.g., hospitalization, absence from home and work, childcare, etc.).	Cost of treatment Cost of absence from home
Accommodation	The extent health providers and their services are organised to fulfil the needs, preferences, and constraints of the patient.	Leaving home for treatment Waiting time for treatment
Acceptability	The patient's views and beliefs concerning health personnel and facilities as well as attitudes of the health personnel towards the patient.	Fear of treatment Lack of information on treatment Use of alternative medicine Trust in health workers

The sample size was planned based on the available patient data for the study timeframe and practical considerations related to data collection. We aimed to include 10 patients for each of the six cancer types per registry, resulting in a target of 60 patients per registry and a total sample size of 660 patients. Local interviewers were provided with a list of randomly selected patients. However, the lack of data for some cancer types led to varying sampling fractions and the exclusion of certain cancer types in some registries (Data S1B).

### 2.3 Data collection

The responsible teams from the cancer registries of the African Cancer Registry Network (AFCRN) conducted the interviews between January 2021 and June 2022. In total, 5955 patients with one of the respective cancer types were registered during the study timeframe, of which 553 questionnaires were included in this study (Data S1A,B). Patients were excluded and replaced if the registry data (or subsequent patient information) indicated the presence of metastases at the time of diagnosis or if the patients were unreachable after three consecutive contact attempts.

Local data collectors contacted patients or their caretakers via telephone. Interviews were conducted with the patients, except if they were deceased or preferred to be represented by a relative. If the patient was unavailable or deceased, caretakers were asked to respond to the questionnaire on behalf of the patient. All participants were asked the full range of questions regarding perceived barriers, regardless of their treatment status for the respective modality. The interviews were documented using EpiData software.^21^

### 2.4 Statistical analysis

**FIGURE 1:**
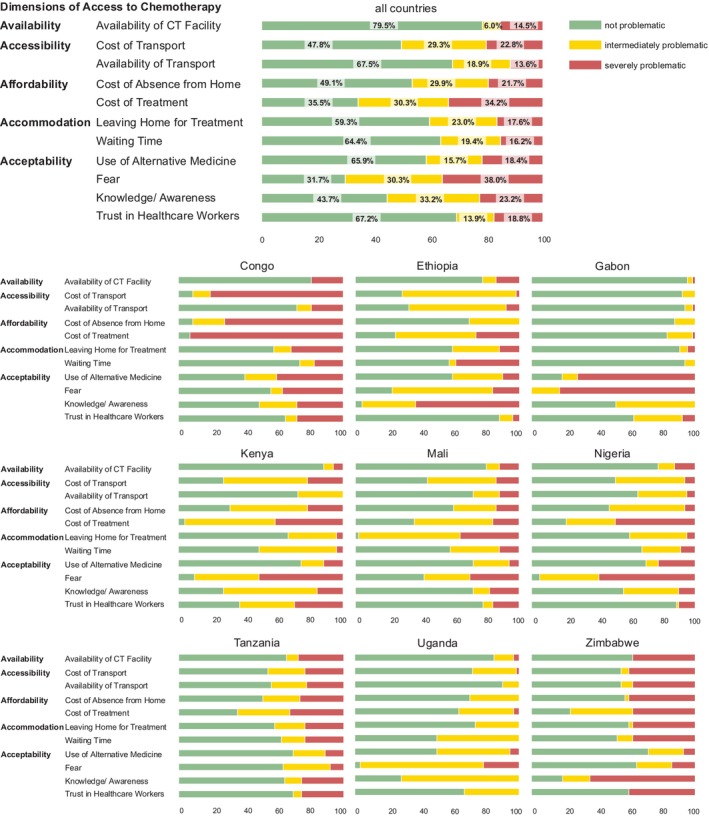
Patient‐reported access to chemotherapy overall and per country. Access to chemotherapy across nine African population‐based cancer registries according to the five dimensions of access to care by Pechansky et al.^18^; barriers are reported as being either not problematic (green), intermediate (yellow), or severely problematic (red), *n* = 553.

All data processing and statistical analyses were carried out using SPSS v23 or the Statsmodels v0.14.1^22^ library for Python. Data cleaning and imputation of missing data were conducted in Python. Imputation was applied to 0.4% of data points in the variable ‘education level’ and 7.2% of data points in the variable ‘self‐perceived wealth’. Due to collinearity, these variables were combined into a single variable (‘education and wealth’). Descriptive analyses were then performed for sociodemographic characteristics, diagnosis, treatment status, and barriers to treatment. We assessed the influence of several patient‐related factors on each of the five dimensions of care using five ordinal logistic regression models. The models were parameterised to determine whether patient‐related factors predicted the ordinal outcomes (‘barriers’). The resulting coefficients were transformed into effect sizes (odds ratios [ORs] and their corresponding 95% confidence intervals [CIs]) (Figure 1). Subsequently, a logistic regression was conducted to assess the likelihood of receiving chemotherapy treatment (binary outcome) based on patient‐related factors (predictors). This analysis included only the subset of patients who reported having chemotherapy treatment recommended to them. The resulting coefficients were also transformed into effect sizes (ORs).

## 3 RESULTS

### 3.1 Patient characteristics

We included 553 interviews. Approximately half of the interviews were conducted with caretakers (54.8%, *n* = 303), while the remainder were conducted with the patients themselves (45.2%, *n* = 250). The highest median age was observed among patients with prostate cancer (71 years), while the lowest was among patients with Kaposi sarcoma (46 years). Of the total cohort, 59.9% were female (*n* = 331), among non‐sex specific cancer (colorectal cancer, NHL, Kaposi Sarcoma), 46.0% were female (97 out of 211). Approximately half of the patients had completed higher education (51.9%), while 12.3% were illiterate. Most participants perceived their own or the patient's wealth as poor (46.3%) or middle class (43.8%).

At the time of the study, three of five patients were alive (61.8%); however, approximately half of the patients with cervical cancer (50.8%) and NHL (50.7%) had died. Chemotherapy was recommended for 71.8% (*n* = 399) of patients, and 58.6% (*n* = 326) received the treatment. Consequently, 13.2% (*n* = 73) were recommended chemotherapy but did not receive it. Table 2 summarises the characteristics of the patient cohort.

**TABLE 2:** Patient characteristics.

Characteristics	All n = 553 (%)	Breast *n* = 116 (%)	Cervix *n* = 118 (%)	CRC *n* = 89 (%)	Prostate *n* = 108 (%)	Kaposi *n* = 49 (%)	NHL *n* = 73 (%)
Age group (years)							
<26	14 (2.5)	1 (0.9)	0	1 (1.1)	0	5 (10.2)	7 (9.6)
26–35	53 (9.6)	15 (12.9)	9 (7.6)	11 (12.4)	0	9 (12.3)	9 (12.3)
36–45	101 (18.3)	32 (27.6)	25 (21.2)	16 (18.0)	0	10 (24.7)	18 (24.7)
46–55	127 (22.7)	33 (28.4)	39 (33.1)	21 (23.6)	3 (2.8)	14 (23.3)	17 (23.3)
56–65	108 (19.5)	17 (14.7)	28 (23.7)	21 (23.6)	29 (26.9)	4 (12.3)	9 (12.3)
66–75	100 (18.1)	13 (11.2)	15 (12.7)	14 (15.7)	45 (41.7)	5 (11.0)	8 (11.0)
75+	50 (9.0)	5 (4.3)	2 (1.7)	5 (5.7)	31 (28.7)	2 (4.1)	5 (6.8)
Median	54.6	50.26	52.8	53.31	70.84	45.81	48.66
Sex							
Female	331 (59.9)	116 (100)	118 (100)	42 (47.2)	0	19 (38.8)	36 (49.3)
Male	222 (40.1)	0	0	47 (52.8)	108 (100)	30 (61.2)	37 (50.7)
Survival status							
Alive	342 (61.8)	82 (70.7)	58 (49.2)	57 (64.0)	73 (67.6)	36 (73.5)	36 (49.3)
Dead	211 (38.2)	34 (29.3)	60 (50.8)	32 (36.0)	35 (32.4)	13 (26.5)	37 (50.7)
Education level							
Cannot read and write	68 (12.3)	14 (12.1)	25 (21.2)	5 (5.6)	13 (12.0)	3 (6.1)	8 (11.0)
Can read and write	78 (14.1)	14 (12.1)	15 (12.7)	16 (18.0)	19 (17.6)	2 (4.1)	12 (16.4)
Grade 1–8	118 (21.3)	21 (18.1)	27 (22.9)	16 (18.0)	19 (17.6)	15 (30.6)	20 (27.4)
Grade 9–12	111 (20.1)	26 (22.4)	23 (19.5)	14 (15.7)	14 (13.0)	16 (32.7)	18 (24.7)
Diploma: college	100 (18.1)	24 (20.7)	13 (11.0)	22 (24.7)	24 (22.2)	10 (20.4)	7 (9.6)
Bachelor degree	58 (10.5)	14 (12.1)	12 (10.2)	11 (12.4)	13 (12.0)	2 (4.1)	6 (8.2)
Master degree	18 (3.3)	3 (2.6)	3 (2.5)	5 (5.6)	5 (4.6)	1 (2.0)	1 (1.4)
NA	2 (0.4)	0	0	0	1 (0.9)	0	1 (1.4)
Self‐perceived wealth							
Very poor	65 (11.8)	12 (10.3)	16 (13.6)	9 (10.1)	13 (12.0)	6 (5.6)	9 (12.3)
Poor	191 (34.5)	50 (43.1)	47 (39.8)	24 (27.0)	30 (27.8)	18 (16.7)	22 (30.1)
Middle Class	242 (43.8)	45 (38.8)	46 (39.0)	47 (52.8)	52 (48.1)	24 (22.2)	28 (38.4)
Rich	15 (2.7)	2 (1.7)	0	3 (3.4)	7 (6.5)	1 (0.9)	2 (2.7)
NA	40 (7.2)	7 (6.0)	9 (7.6)	6 (6.7)	6 (5.6)	0	12 (16.4)
Chemotherapy: treatment status							
Received	326 (58.6)	83 (71.6)	59 (50.0)	63 (70.8)	34 (31.5)	35 (32.4)	52 (71.2)
Recommended but not received	73 (13.2)	17 (14.7)	25 (21.2)	14 (15.7)	6 (5.6)	2 (1.9)	9 (12.3)
Not Recommended	154 (27.8)	16 (13.8)	34 (28.8)	12 (13.5)	68 (63.0)	12 (11.1)	12 (16.4)

Abbreviations: Kaposi, Kaposi Sarcoma; NA, not answered; NHL, non‐Hodgkin lymphoma.

### 3.2 Barriers to treatment

#### 3.2.1 Top overall barriers to receiving chemotherapy across all registries

Most patients reported fear as their greatest concern (70.2% impeding, comprising intermediate and severely problematic; 35.8% severely problematic), followed by the cost of treatment (65.7%, 33.6%), knowledge/awareness about the treatment (55.4%, 22.5%), cost of transport (50.3%, 20.2%), and costs associated with being absent from home (46.5%, 19.4%).

### 3.3 Barriers to receiving chemotherapy by country

Fear of treatment was the most common barrier overall and ranked among the top three barriers in six of nine countries (Figure 1). In Gabon, all patients identified fear as an impeding factor, with 82.8% describing it as severely problematic. In Nigeria, Uganda, and Kenya, more than 90% of patients reported fear as impeding (95.0%, 96.5%, and 90.2%, respectively). In the Republic of Congo, all patients rated the cost of treatment as severely problematic. Similarly, the cost of treatment was the main barrier in Kenya (96.0% impeding, 41.1% severely problematic) and Tanzania (64.3%, 32.7%). Lack of knowledge or awareness about treatment was the leading barrier for patients in Ethiopia (95.9%, 63.3%) and Zimbabwe (80.9%, 64.3%), although it was less commonly reported in Mali (28.0%). In Mali, nearly all participants (98.0%) identified leaving home for treatment as a impeding. In Zimbabwe (38.1%) and Tanzania (34.7%), more than one‐third of participants reported the availability of health facilities as an impeding factor. The availability of transportation was frequently considered impeding by patients in Ethiopia (67.4%). Waiting times were a common issue across most countries and were considered impeding by approximately half of the participants in Kenya (51.0%), Uganda (50.0%), and Zimbabwe (47.6%). Overall, 69.3% of patients reported no issues with trust in health professionals. However, in Kenya and Zimbabwe, a higher proportion of patients perceived a lack of trust in health professionals as impeding (62.7% and 29.4%; 40.5% and 40.5%, respectively). The use of alternative medicine as a substitute for chemotherapy was reported as impeding by patients in Gabon (81.3%, 71.9%) and the Republic of Congo (59.7%, 40.4%) (Data S1C,D).

Higher levels of education and wealth were associated with fewer problems related to the availability of transport to chemotherapy (OR, 0.62; 95% CI, 0.50–0.76), leaving home for therapy (OR, 0.74; 95% CI, 0.62–0.88), waiting time (OR, 0.82; 95% CI, 0.68–0.97), cost of treatment (OR, 0.65; 95% CI, 0.56–0.77), cost of transport (OR, 0.61; 95% CI, 0.51–0.73), and cost of being absent from home (OR, 0.73; 95% CI, 0.62–0.86). However, these factors were associated with increased problems related to fear of treatment (OR, 1.29; 95% CI, 1.11–1.50) (Table 3). A higher country human development index (HDI) was linked to fewer problems with transport to chemotherapy (OR, 0.68; 95% CI, 0.52–0.90), leaving home for therapy (OR, 0.40; 95% CI, 0.31–0.54), waiting time (OR, 0.63; 95% CI, 0.49–0.82), and cost of treatment (OR, 0.76; 95% CI, 0.61–0.96). However, a higher HDI was associated with increased challenges related to fear of treatment (OR, 1.98; 95% CI, 1.56–2.51), lack of trust in health professionals (OR, 1.63; 95% CI, 1.27–2.09), and the use of alternative medicine (OR, 2.79; 95% CI, 2.14–3.63). Caretaker responses were associated with increased problems regarding cost of absence from home (OR, 1.47; 95% CI, 1.03–2.10). Kaposi sarcoma was associated with more significant problems, including the cost of transport and the cost of being absent from home (OR, 0.30; 95% CI, 0.14–0.63; OR, 0.42; 95% CI, 0.20–0.88). A sensitivity analysis of patient subgroups based on their treatment status (received, not received, recommended, not recommended) did not reveal distinct results, although observed trends were comparable to those in the full cohort (Data S1F).

**TABLE 3:** Associations with barrier report.

Variable	Availability	Accessibility		Affordability		Accommodation		Acceptability			
	Availability of CT‐Facility	Availability of Transport	Cost of Transport	Cost of Treatment	Cost of Absence from Home	Leaving Home for Treatment	Waiting Time	Fear	Lack of Information	Trust in Health Workers	Use of Alternative Medicine
	OR (95%‐CI)	OR (95%‐CI)	OR (95%‐CI)	OR (95%‐CI)	OR (95%‐CI)	OR (95%‐CI)	OR (95%‐CI)	OR (95%‐CI)	OR (95%‐CI)	OR (95%‐CI)	OR (95%‐CI)
Education & wealth (from 0 to 6), *n* = 550											
Higher Education & Wealth	0.82 (0.66; 1.01)	**0**.**62** (0.50; 0.76)	**0**.**61** (0.51; 0.73)	**0**.**65** (0.56; 0.77)	**0**.**73** (0.62; 0.86)	**0**.**74** (0.62; 0.88)	**0**.**82** (0.68; 0.97)	**1**.**29** (1.11; 1.50)	0.94 (0.81; 1.09)	0.90 (0.75; 1.07)	0.90 (0.76; 1.07)
HDI (from 0.43 to 0.71), *n* = 550											
Higher HDI (steps of 0.1)	0.79 (0.59; 1.07)	**0**.**68** (0.52; 0.9)	0.79 (0.62; 1.0)	**0**.**76** (0.61; 0.96)	0.97 (0.77; 1.22)	**0**.**40** (0.31; 0.54)	**0**.**63** (0.49; 0.82)	**1**.**98** (1.56; 2.51)	0.97 (0.78; 1.21)	**1**.**63** (1.27; 2.09)	**2**.**79** (2.14; 3.63)
Responding person (self*, *n* = 241)											
Other, *n* = 309	1.41 (0.89; 2.23)	0.94 (0.63; 1.4)	1.34 (0.94; 1.92)	1.42 (1.0; 2.01)	**1**.**47** (1.03; 2.1)	1.23 (0.84; 1.79)	1.11 (0.76; 1.63)	1.22 (0.86; 1.72)	1.03 (0.73; 1.45)	1.18 (0.79; 1.77)	1.22 (0.83; 1.78)
Sex (Female*, *n* = 329)											
Male, *n* = 221	0.52 (0.24; 1.12)	0.92 (0.49; 1.74)	1.23 (0.71; 2.12)	1.42 (0.85; 2.36)	1.36 (0.8; 2.33)	1.13 (0.63; 2.03)	1.00 (0.56; 1.79)	**0**.**60** (0.36; 0.99)	0.72 (0.44; 1.18)	1.22 (0.66; 2.27)	0.89 (0.51; 1.56)
Age, *n* = 550											
Higher Age	1.00 (0.98; 1.01)	0.99 (0.98; 1.01)	0.99 (0.98; 1.0)	0.99 (0.98; 1.01)	0.99 (0.98; 1.01)	0.99 (0.98; 1.01)	1.00 (0.98; 1.01)	1.00 (0.98; 1.01)	1.00 (0.99; 1.01)	1.00 (0.98; 1.01)	1.00 (0.99; 1.02)
Entity (NHL*, *n* = 72)											
Breast, *n* = 115	1.04 (0.47; 2.32)	0.97 (0.48; 1.95)	0.94 (0.5; 1.75)	1.44 (0.78; 2.68)	1.07 (0.57; 1.99)	1.46 (0.74; 2.86)	1.13 (0.56; 2.26)	0.90 (0.49; 1.68)	0.75 (0.41; 1.37)	1.39 (0.66; 2.91)	0.74 (0.38; 1.45)
Cervix, n = 118	0.89 (0.40; 1.96)	0.68 (0.34; 1.37)	0.66 (0.36; 1.24)	1.30 (0.71; 2.40)	0.73 (0.39; 1.35)	0.93 (0.47; 1.83)	1.31 (0.67; 2.58)	1.37 (0.74; 2.53)	0.64 (0.35; 1.16)	1.08 (0.52; 2.25)	1.43 (0.75; 2.74)
Colorectum, *n* = 89	0.86 (0.37; 1.97)	0.53 (0.26; 1.07)	0.56 (0.31; 1.03)	1.11 (0.61; 2.01)	0.71 (0.39; 1.30)	0.92 (0.47; 1.80)	1.30 (0.67; 2.5)	1.14 (0.63; 2.06)	0.72 (0.41; 1.28)	1.02 (0.50; 2.07)	0.95 (0.50; 1.8)
Prostate *n* = 107	2.54 (1.01; 6.39)	1.59 (0.75; 3.38)	1.18 (0.59; 2.35)	0.99 (0.50; 1.97)	0.91 (0.45; 1.83)	1.41 (0.68; 2.95)	2.62 (1.25; 5.47)	1.35 (0.68; 2.68)	1.39 (0.71; 2.70)	2.16 (1.00; 4.69)	1.25 (0.61; 2.57)
Kaposi, *n* = 49	0.56 (0.19; 1.72)	**0**.**38** (0.15; 0.95)	**0**.**30** (0.14; 0.63)	0.52 (0.26; 1.04)	**0**.**42** (0.20; 0.88)	0.77 (0.35; 1.7)	0.73 (0.32; 1.69)	0.78 (0.39; 1.56)	0.76 (0.39; 1.49)	0.69 (0.30; 1.60)	0.71 (0.33; 1.50)

*Note*: Multivariable ordinal regression reporting odds ratios (adjusted for all items mentioned) for factors influencing barriers to care. Odds ratios >1 indicate increased probability of increased problem report. *reference category. Bold values indicate odds ratios with 95% confidence intervals excluding 1.

Abbreviations: CI, confidence interval; HDI, human development index of the country; NHL, non‐Hodgkin lymphoma; OR, odds ratio; CT, chemotherapy.

### 3.4 Barriers to receiving chemotherapy stratified by receipt of treatment

Of the patients who reported being recommended chemotherapy, 81.7% received it (Table 4). Regardless of treatment recommendation or receipt, the cost of treatment was the most commonly reported severe barrier. Fear was identified as the most impeding barrier by patients who were recommended but did not receive treatment (84.9%) and by those who received treatment (73.9%).

**TABLE 4:** access‐report according to chemotherapy treatment status (not received, recommended but not received, received).

		Total (*n* = 551)	Not recommended (*n* = 152; 27.6%)	Recommended but not received (*n* = 73; 13.2%)	Received (*n* = 326; 59.2%)			
		*n* (%)	*n* (%)	*n* (%)	*n* (%)	Not recommended	Recommended not received	Received
*Availability*								
Availability of chemotherapy facility	**Severe**	83 (15.1)	49 (32.2)	13 (17.8)	21 (6.5)	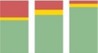		
	**Intermediate**	35 (6.4)	8 (5.3)	7 (9.6)	20 (6.2)			
	**Not problematic**	423 (78.5)	95 (62.5)	53 (72.6)	284 (87.4)			
*Accessibility*								
Cost of transport	**Severe**	111 (20.1)	59 (38.8)	13 (17.8)	39 (12.0)	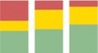		
	**Intermediate**	166 (30.1)	29 (19.1)	27 (37.0)	110 (33.7)			
	**Not problematic**	274 (49.7)	64 (42.1)	33 (45.2)	177 (54.3)			
Availability of transport	**Severe**	64 (11.6)	45 (29.6)	9 (12.3)	10 (3.1)	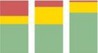		
	**Intermediate**	113 (20.5)	23 (15.1)	24 (32.9)	66 (20.2)			
	**Not problematic**	374 (67.9)	84 (55.3)	40 (54.8)	250 (76.7)			
*Affordability*								
Cost of treatment	**Severe**	185 (33.6)	67 (44.1)	36 (49.3)	82 (25.2)	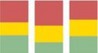		
	**Intermediate**	177 (32.1)	33 (21.7)	25 (34.2)	119 (36.5)			
	**Not problematic**	189 (34.5)	52 (34.2)	12 (16.4)	125 (38.3)			
Cost of absence from home	**Severe**	107 (19.4)	60 (39.5)	11 (15.1)	36 (11.0)	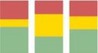		
	**Intermediate**	149 (27.0)	21 (13.8)	30 (41.1)	98 (30.1)			
	**Not problematic**	295 (53.5)	71 (46.7)	32 (43.8)	192 (58.9)			
*Accommodation*								
Leaving home for treatment	**Severe**	90 (16.3)	49 (32.2)	10 (13.7)	31 (9.5)	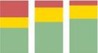		
	**Intermediate**	132 (24.0)	30 (19.7)	20 (27.4)	82 (25.2)			
	**Not problematic**	329 (59.7)	73 (48.0)	43 (58.9)	213 (36.5)			
Waiting time	**Severe**	83 (15.1)	64 (42.1)	11 (15.1)	8 (2.5)	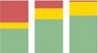		
	**Intermediate**	117 (21.2)	17 (11.2)	15 (20.5)	85 (26.1)			
	**Not problematic**	351 (63.7)	71 (46.7)	47 (64.4)	233 (71.5)			
*Acceptability*								
Use of alternative medicine	**Severe**	118 (21.7)	43 (28.7)	19 (26.0)	56 (17.4)	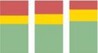		
	**Intermediate**	108 (19.8)	24 (16.0)	14 (19.2)	70 (21.7)			
	**Not problematic**	319 (58.5)	83 (55.3)	40 (54.8)	196 (60.9)			
Fear	**Severe**	197 (35.8)	56 (36.8)	30 (41.1)	111 (34.0)	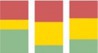		
	**Intermediate**	190 (34.5)	28 (18.4)	32 (43.8)	130 (39.9)			
	**Not problematic**	164 (29.8)	68 (44.7)	11 (15.1)	85 (26.1)			
Knowledge/awareness	**Severe**	124 (22.5)	71 (46.7)	12 (16.4)	41 (12.6)	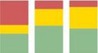		
	**Intermediate**	181 (32.8)	25 (16.4)	23 (31.5)	133 (40.8)			
	**Not problematic**	246 (44.6)	56 (36.8)	38 (52.1)	152 (46.6)			
Trust in health workers	**Severe**	96 (17.4)	55 (36.2)	13 (17.8)	28 (8.6)	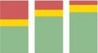		
	**Intermediate**	73 (13.2)	19 (12.5)	9 (12.3)	45 (13.8)			
	**Not problematic**	382 (69.3)	78 (51.3)	51 (69.9)	253 (77.6)			

### 3.5 Factors associated with probability of receiving chemotherapy (considering only patients with hemotherapy ‘recommended and received’ or ‘recommended and not received’)

**FIGURE 2:**
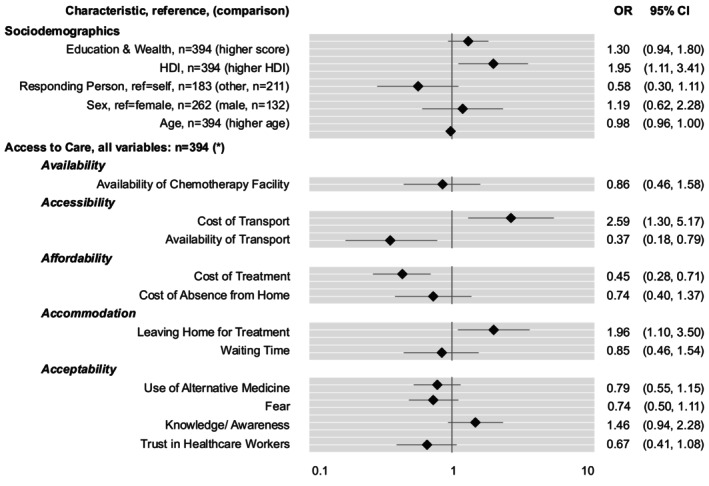
Associations with (self‐reported) receipt of chemotherapy. Odds ratios >1 indicate an increased probability of receiving chemotherapy. Only patients for whom chemotherapy had been recommended (*n* = 395) were included in the analysis. *, increasingly problematic; HDI, human development index; OR, odds ratio.

Being recommended chemotherapy and perceiving the availability of transport and the cost of treatment as more problematic were associated with a decreased likelihood of receiving treatment (OR, 0.37; 95% CI, 0.18–0.79 and OR, 0.45; 95% CI, 0.28–0.71). Conversely, a higher HDI was associated with an increased likelihood of receiving treatment (OR, 1.95; 95% CI, 1.11–3.41). Interestingly, perceiving the cost of transport and leaving home for treatment as more problematic was linked to an increased likelihood of receiving treatment (OR, 2.59; 95% CI, 1.30–5.17 and OR, 1.96; 95% CI, 1.10–3.50) (Figure 2).

## 4 DISCUSSION

While chemotherapy is an essential component of cancer care, participants in our study reported substantial barriers to accessing treatment. Patients from different countries highlighted challenges across various categories of barriers to care, as defined by Penchansky and Thomas.^20^ Many participants perceived barriers related to acceptability, affordability, and accessibility.

### 4.1 Acceptability

The acceptability of chemotherapy was a major issue for patients with cancer in SSA. Problems related to fear of treatment and knowledge or awareness were widely reported across most countries. Interestingly, higher levels of education and wealth were associated with a greater perception of fear. Patients who reported fear of treatment tended to receive less chemotherapy. Findings from another study in South Africa indicated that fear of adverse or undesirable effects of chemotherapy hindered treatment uptake among patients with breast cancer.^23^ Several studies from Kenya identified a lack of patient knowledge about their respective cancer as a key factor for not seeking treatment. This was noted particularly in studies focusing on cervical cancer^19^, ^24^ and other cancer types.^6^, ^18^, ^19^, ^24^ Providing detailed information about the treatment plan, enhancing emotional support networks, and adopting a multidisciplinary approach—including psychosocial counselling and psychological support—seems to contribute to the healing process and improve the quality of life for patients with cancer.^25^, ^26^ Replacing chemotherapy with alternative medicine was reported by a significant proportion of patients in some countries (e.g., Gabon, the Republic of the Congo, and Uganda). The use of alternative medicine (including traditional and complementary therapies) in SSA is extensive and often underreported,^27^ and it represents a common barrier to receiving timely, guideline‐concordant treatment.^28^, ^29^, ^30^ While a lack of trust in health workers was less frequently reported than other barriers, studies from Zimbabwe (citing ‘bad attitudes of health workers’),^19^ Ghana, and Mali (highlighting a lack of trust in community health centres) identified it as a relevant issue.^28^, ^31^ This is particularly concerning because healthcare workers are the primary source of information and play a crucial role in addressing acceptability issues.^32^

### 4.2 Affordability (cost of treatment, cost of being absent from home)

High costs are reportedly the main reason for not starting treatment after diagnosis^6^, ^18^ and for the early interruption of chemotherapy.^33^, ^34^ Our study demonstrated that the cost of treatment was considered a major barrier among patients in most of the countries involved. Although the World Health Organization included several chemotherapeutic drugs in its list of essential medicines in 2015, they remain unaffordable for many patients in LMICs, even in regions with universal health coverage.^5^, ^35^ Free treatment can improve adherence rates, as shown in studies from Kenya^24^ and Namibia.^13^ However, despite chemotherapy being offered free of charge in Tanzania, the cost of treatment was still reported as a major barrier in our study. A likely explanation is that diagnostic and other hospitalisation costs still need to be paid by patients.^36^ Additionally, when chemotherapy drugs are out of stock, patients may need to purchase expensive alternatives from non‐public sources. Similarly, in Malawi, patients reported that while the cost of chemotherapy is partially covered in the public sector, shortages often require purchases from private pharmacies, resulting in substantial financial burdens.^36^ In Gabon, where free cancer treatment is provided, only a small number of patients reported affordability issues. Finally, patients with Kaposi sarcoma perceived costs as less problematic, likely because of the availability of domestic and international HIV patient support programmes.^37^

Affordability is a pivotal aspect of access to chemotherapy. Our findings indicate that affordability issues were a major factor hindering treatment uptake, highlighting the need for financial support and an expansion of health insurance coverage, particularly for socioeconomically disadvantaged patients. Studies have suggested increased utilisation of endocrine therapies, such as tamoxifen, for patients with breast cancer because it is available at low cost.^5^, ^38^ This approach might enhance treatment efficiency without imposing additional financial burdens on patients and is already included in existing context‐specific and cost‐adapted treatment protocols.

### 4.3 Accessibility (accessibility of transport, cost of transport)

Overall, public transport was considered available by approximately three‐quarters of the patients, although half of them regarded the costs as problematic. Most registries are located in urban centres, where public transport is generally more accessible compared with rural areas.^39^ Literature from SSA has indicated that a lack of public transport and the cost of transport can delay treatment for patients with cancer.^24^, ^34^, ^40^, ^41^ For example, women from Malawi reported saving money for 3 months to afford transportation to treatment.^36^ Interestingly, patients who reported issues with the affordability of transport were more likely to have adhered to chemotherapy. This association may suggest that after completing six or eight courses of chemotherapy, the cumulative costs of transport became considerable and were retrospectively identified as problematic. We recommend integrating transportation plans into cancer health service planning to address these barriers effectively.^18^

### 4.4 Availability (availability of health centre)

In our study, availability was largely perceived as unproblematic by patients. Nevertheless, the literature highlights a challenging situation, including a lack of trained health professionals to administer chemotherapy and the unavailability of specific drugs.^42^ Studies have shown that shortages of chemotherapeutic agents and supply stockouts frequently result in treatment delays or interruptions.^6^, ^24^, ^33^, ^42^, ^43^ Additionally, tertiary medical cancer care resources in SSA are often concentrated in urban areas, leaving rural dwellers at practical and financial disadvantages. Rural health centres frequently lack the necessary resources for cancer treatment.^10^, ^36^ Even in urban settings, a lack of specialised health centres remains a significant issue.^31^

### 4.5 Accommodation (waiting time, leaving home)

Leaving home to receive chemotherapy was a major problem for patients in Mali, in contrast to all other countries. Although participants were not asked to explain their responses, another study from Mali suggested that a lack of social support might contribute to this issue.^31^ Some patients reported waiting times as problematic, particularly in Uganda, Zimbabwe, and Kenya. Several studies have highlighted issues with supply stockouts in Kenya, which may contribute to these prolonged waiting times.^6^, ^24^ Moreover, chemoradiation treatment schemes are sometimes postponed because of insufficient radiotherapy capacities. However, studies have suggested prioritising chemotherapy when delays with planned radiotherapy occur.^44^

## 5 STRENGTHS AND LIMITATIONS

The information collected for this study reflects the subjective views of patients or their caretakers and is limited to their knowledge. Additional data from patient files to verify chemotherapy recommendations or receipt were not available. Consequently, we chose to assess the frequency of perceived barriers and their associated predictors across all patients, regardless of self‐reported treatment recommendations or receipt. The use of different interviewers in each country—and sometimes even within the same country—may have introduced bias. We minimised this risk through comprehensive training for data collectors. Additionally, because most registries included in this study are located in larger cities, our sample was primarily composed of urban inhabitants and is therefore not representative of the entire population in these countries. Also, this study included patients diagnosed between January 2018 and December 2019 to exclude those diagnosed during the COVID‐19 pandemic because they likely faced a unique and complex set of barriers compared to patients diagnosed before or after the pandemic. Finally, to mitigate survivorship bias, we included patients' caretakers. We adjusted their responses in the regression analyses to assess differences between patient and caretaker responses.

## 6 CONCLUSION

This population‐based, multi‐country study revealed that chemotherapy was available to more than six in seven patients from urban population‐based cancer registries. However, a complex set of barriers to accessing chemotherapy remains prevalent across SSA, particularly regarding affordability, accessibility, and acceptability of treatment. Differences in the perception of barriers were influenced by patient characteristics such as wealth, education, and country HDI. Patients with greater wealth and higher levels of education perceived fewer barriers, except for fear of treatment. By contrast, type of cancer, age, and sex were less associated with perceived barriers to care. To effectively target and mitigate barriers to accessing chemotherapy, tailored approaches are necessary. These should account for socioeconomic differences and incorporate social support structures to address concerns and fears related to treatment. We anticipate that such measures would increase the number of patients with cancer receiving guideline‐concordant treatment with chemotherapeutic agents, thereby improving survival rates.

## AUTHOR CONTRIBUTIONS

**Tamara König:** Conceptualization; funding acquisition; writing – original draft; methodology; writing – review and editing; visualization; formal analysis; project administration; data curation. **Nikolaus Christian Simon Mezger:** Methodology; data curation; writing – review and editing; study conceptualization; supervision; investiagation: project administration. **Ole Stoeter:** Conceptualization; writing – review and editing; visualization; methodology. **Phoebe Mary Amulen:** Investigation; writing – review and editing. **Margaret Borok:** Investigation; writing – review and editing. **Gladys C. Chesumbai:** Investigation; writing – review and editing. **Moudiongui MBoungou Dimitry:** Investigation; writing – review and editing. **Ima‐Obong Ekanem:** Investigation; writing – review and editing. **Adugna Fekadu:** Writing – review and editing. **Bakarou Kamaté:** Writing – review and editing; investigation. **William Muller:** Investigation; writing – review and editing. **Alex Alain Kabena Nzambikolo:** Writing – review and editing; investigation. **Abidemi Omonisi:** Investigation; writing – review and editing. **Furaha Serventi:** Investigation; writing – review and editing. **Markus Wallwiener:** Conceptualization; funding acquisition; writing – review and editing. **Liu Biying:** Conceptualization; funding acquisition; methodology; project administration; supervision; writing – review and editing. **Donald Maxwell Parkin:** Funding acquisition; conceptualization; project administration; writing – review and editing. **Pablo Sandro Carvalho Santos:** Formal analysis; software; supervision; data curation; resources; visualization; methodology; validation; writing – review and editing. **Eva Johanna Kantelhardt:** Supervision; resources; project administration; methodology; validation; visualization; writing – review and editing; funding acquisition; conceptualization. **Eric Sven Kroeber:** Writing – review and editing; visualization; methodology; formal analysis; project administration; supervision.

## FUNDING INFORMATION

This work was supported by

1. German Federal Ministry of Education and Research (01KA2220B; recipient: Eva Johanna Kantelhardt).
2. Else Kroener‐Fresenius‐Foundation (2018_HA31SP; recipient: Eva Johanna Kantelhardt)
3. Science for Africa Foundation under its Developing Excellence in Leadership, Training and Science in Africa (DELTAS Africa) program (Del‐22‐008; recipient: Eva Johanna Kantelhardt) with support from the Wellcome Trust and the UK Foreign, Commonwealth & Development Office and is part of the EDCPT2 program supported by the European Union

## CONFLICT OF INTEREST STATEMENT

We have no conflicts of interest to disclose.

## ETHICS STATEMENT

The AFCRN research committee and the Martin Luther University Halle‐Wittenberg, Germany approved this study (3 December 2020, reference: 2020‐192). Local ethical clearance was obtained from responsible authorities for the Ekiti (reference: EKSUTH/A67/2021/02/006) and Calabar (reference: UCTH/HREC/33/118) registries. For the other registries, further ethical approval was not requested by the research committees of the countries concerned since secondary data were used, interviews were done during routine registry data collection, and results were fully anonymized. Oral consent for participation was obtained during the interviews and documented on the questionnaires.

## Supporting information


**DATA S1.** Supporting Information.

## Data Availability

The data that support the findings of this study and further information are available from the corresponding author upon request.
