# Supplementary material for: Patient‐ and caregiver‐reported barriers to chemotherapy in nine sub‐Saharan African countries: A cross‐sectional survey among population‐based registries
Source: Int J Cancer. 2025 Dec 30;158(10):2684–96. doi: 10.1002/ijc.70309 (PMC12996735; doi:10.1002/ijc.70309)
Supplement: Supplementary file 1 — DATA S1. Supporting Information. [file IJC-158-2684-s001.pdf]

Patient- and Caregiver-reported barriers to chemotherapy in nine Sub-Saharan African countries – a cross-sectional survey among population-based registries - Supplement

Tamara König, M.D., Nikolaus Christian Simon Mezger, M.D. , Ole Stoeter, M.D., Phoebe Mary Amulen, B.A., Margaret Borok, M.D., Gladys C. Chesumbai, M.Sc., Moudiongui MBoungou Dimitry, Ima-Obong Ekanem, M.D., Adugna Fekadu, MD, Bakarou Kamaté, M.D., William Muller, M.D., Alex Alain Kabena Nzambikolo, M.Sc, Abidemi Omonisi, M.D., , Furaha Serventi, M.D., Markus Wallwiener, Biying Liu, M.Sc., Donald Maxwell Parkin, M.D. , Pablo Sandro Carvalho Santos, Ph.D., Eva Johanna Kantelhardt, M.D., Eric Sven Kroeber, M. D.

Table of contents:

Supplement A - Flow chart of the cohort
Supplement B - Countries, Registries and Cancer Patients
Supplement C1 - Reported access to chemotherapy items per country
Supplement C2 - Reported access to Chemotherapy items per country
Supplement D1 - Reported access to Chemotherapy items per cancer entity
Supplement D2 - Reported access to Chemotherapy items per cancer entity
Supplement E - Questionnaire items - Access to Chemotherapy
Supplement F - Associations with barrier report of participants who were recommended chemotherapy

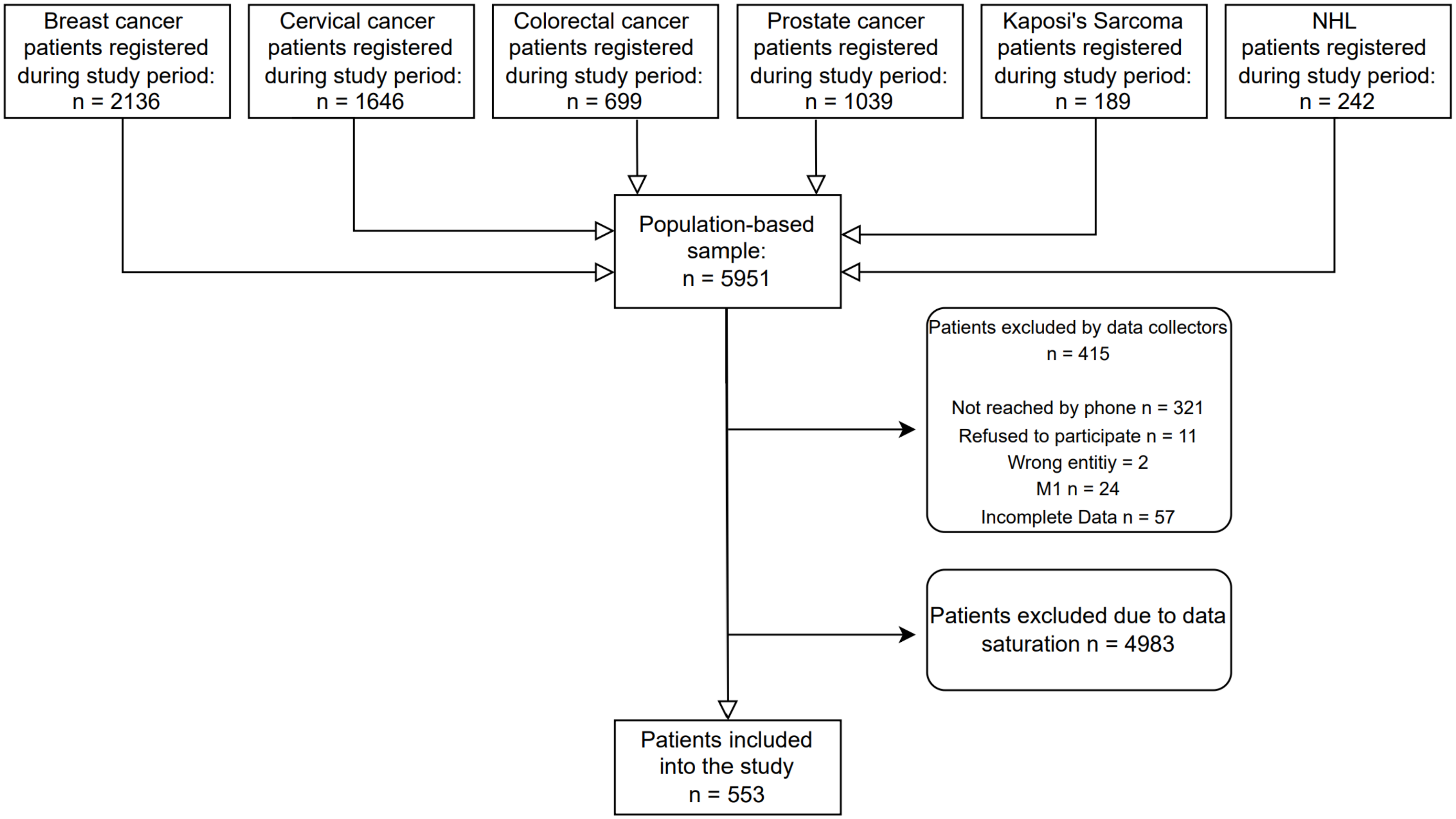

Supplement A Flow chart of the cohort

M1, patient had metastases

Country	City Registry	Country's HDI (rank)	Country MV-ratio	Period of diagnosis	Breast n (n)	Cervix n (n)	Colorectal n (n)	Prostate n (n)	Kaposi n (n)	NHL n (n)	Total n (n)
Rep. of the Congo	Brazzaville	0.57 (low)	0.00	2019	11 (186)	11 (101)	13 (43)	10 (130)	0 (3)	12 (26)	57 (489)
Ethiopia	Addis Abeba	0.50 (low)	0.01	2019	10 (501)	10 (186)	10 (206)	10 (72)	0 (5)	9 (36)	49 (1006)
Gabon	Libreville	0.71 (high)	1.13	2019; NHL + Sarcoma 2018-2019	12 (143)	11 (84)	13 (40)	10 (45)	9 (13)	9 (12)	64 (337)
Kenya	Eldoret	0.60 (medium)	0.25	2018-2019	8 (43)	10 (33)	5 (19)	13 (40)	8 (16)	7 (25)	51 (176)
Mali	Bamako	0.43 (low)	0.07	2018-2019	11 (641)	10 (631)	9 (228)	10 (142)	0 (6)	10 (81)	50 (1729)
Nigeria	Calabar	0.51 (low)	0.08	2019; NHL+CRC 2018-2019	10 (169)	9 (51)	0 (24)	3 (81)	0 (6)	0 (14)	22 (345)
Nigeria	Ekiti	0.51 (low)	0.08	2018-2019	15 (172)	15 (29)	14 (40)	16 (167)	0 (0)	0 (0)	60 (408)
Tanzania	Mbeya	0.55 (low)	0.16	2019; NHL+CRC 2018-2019	10 (30)	12 (109)	6 (17)	6 (17)	10 (24)	6 (12)	50 (209)
Tanzania	Kilimanjaro	0.55 (low)	0.16	2019	9 (94)	10 (131)	9 (41)	10 (172)	0 (10)	10 (20)	49 (468)
Uganda	Kampala	0.53 (low)	0.03	2019	10 (67)	10 (125)	10 (31)	10 (62)	10 (59)	10 (13)	60 (357)
Zimbabwe	Harare	0.60 (medium)	0.38	2018-2019; Kaposi 2019	10 (95)	10 (155)	0 (NA)	10 (138)	12 (43)	0 (NA)	42 (431)
**Total**	*n.a.*	*n.a.*	*n.a.*		116 (2141)	118 (1635)	89 (689)	108 (1066)	49 (185)	73 (239)	553 (5955)

Supplement B Countries, Registries and Cancer Patients

Participants and corresponding entities in each cancer registry (study time frame); CRC, colorectal cancer; HDI, Human Development Index (2019); Kaposi, Kaposi sarcoma; MV-ratio, ratio of megavoltage units per 1000 cancer cases in 2020 (Elmore et al., 2021); n, patients included in the study; (n), patients registered in the cancer registry in the study time frame; NA, not assessed; NHL, non-Hodgkin lymphoma; Rep., Republic

		All countries		Congo		Ethiopia		Gabon		Kenya		Mali (n=50)		Nigeria		Tanzania		Uganda		Zimbabwe	
		n	%	n	%	n	%	n	%	n	%	n	%	n	%	n	%	n	%	n	%
**Availability of Chemotherapy Facility**	**Very**	83	15,1%	11	19,3%	7	14,3%	1	1,6%	3	5,9%	6	12,0%	10	12,5%	27	27,6%	2	3,4%	16	38,1%
	**Problematic**	35	6,4%	0	0,0%	4	8,2%	2	3,1%	3	5,9%	4	8,0%	8	10,0%	7	7,1%	7	11,9%	0	0,0%
	**Not**	432	78,5%	46	80,7%	38	77,6%	61	95,3%	45	88,2%	40	80,0%	62	77,5%	64	65,3%	50	84,7%	26	61,9%
**Cost of Transport**	**Very**	111	20,1%	46	80,7%	1	2,0%	0	0,0%	11	21,6%	7	14,0%	5	6,3%	23	23,5%	1	1,7%	17	40,5%
	**Problematic**	166	30,1%	6	10,5%	34	69,4%	5	7,8%	26	51,0%	21	42,0%	34	42,5%	22	22,4%	16	26,7%	2	4,8%
	**Not**	274	49,7%	5	8,8%	14	28,6%	59	92,2%	14	27,5%	22	44,0%	41	51,3%	53	54,1%	43	71,7%	23	54,8%
**Availability of Transport**	**Very**	64	11,6%	11	19,3%	4	8,2%	1	1,6%	0	0,0%	6	12,0%	4	5,0%	22	22,4%	0	0,0%	16	38,1%
	**Problematic**	113	20,5%	5	8,8%	29	59,2%	3	4,7%	14	27,5%	8	16,0%	24	30,0%	21	21,4%	6	10,0%	3	7,1%
	**Not**	374	67,9%	41	71,9%	16	32,7%	60	93,8%	37	72,5%	36	72,0%	52	65,0%	55	56,1%	54	90,0%	23	54,8%
**Cost of Absence from Home**	**Very**	107	19,4%	41	71,9%	0	0,0%	0	0,0%	11	21,6%	7	14,0%	5	6,3%	26	26,5%	0	0,0%	17	40,5%
	**Problematic**	149	27,0%	11	19,3%	15	30,6%	8	12,5%	24	47,1%	13	26,0%	37	46,3%	22	22,4%	18	30,0%	1	2,4%
	**Not**	295	53,5%	5	8,8%	34	69,4%	56	87,5%	16	31,4%	30	60,0%	38	47,5%	50	51,0%	42	70,0%	24	57,1%
**Cost of Treatment**	**Very**	185	33,6%	53	93,0%	13	26,5%	1	1,6%	21	41,2%	8	16,0%	39	48,8%	32	32,7%	2	3,3%	16	38,1%
	**Problematic**	177	32,1%	0	0,0%	24	49,0%	10	15,6%	28	54,9%	24	48,0%	24	30,0%	31	31,6%	20	33,3%	16	38,1%
	**Not**	189	34,3%	4	7,0%	12	24,5%	53	82,8%	2	3,9%	18	36,0%	17	21,3%	35	35,7%	38	63,3%	10	23,8%
**Leaving Home for Treatment**	**Very**	90	16,3%	18	31,6%	6	12,2%	3	4,7%	2	3,9%	18	36,0%	4	5,0%	23	23,5%	0	0,0%	16	38,1%
	**Problematic**	132	24,0%	6	10,5%	14	28,6%	3	4,7%	15	29,4%	31	62,0%	28	35,0%	18	18,4%	16	26,7%	1	2,4%
	**Not**	329	59,7%	33	57,9%	29	59,2%	58	90,6%	34	66,7%	1	2,0%	48	60,0%	57	58,2%	44	73,3%	25	59,5%
**Waiting Time**	**Very**	83	15,1%	10	17,5%	19	38,8%	0	0,0%	2	3,9%	6	12,0%	7	8,8%	23	23,5%	0	0,0%	16	38,1%
	**Problematic**	117	21,2%	5	8,8%	2	4,1%	4	6,3%	24	47,1%	15	30,0%	19	23,8%	14	14,3%	30	50,0%	4	9,5%
	**Not**	351	63,7%	42	73,7%	28	57,1%	60	93,8%	25	49,0%	29	58,0%	54	67,5%	61	62,2%	30	50,0%	22	52,4%
**Use of Alternative Medicine**	**Very**	118	21,7%	23	40,4%	5	10,2%	46	71,9%	6	11,8%	3	6,0%	18	22,5%	11	11,2%	3	5,6%	3	7,1%
	**Problematic**	108	19,8%	11	19,3%	15	30,6%	6	9,4%	7	13,7%	11	22,0%	6	7,5%	19	19,4%	24	44,4%	9	21,4%
	**Not**	319	58,5%	23	40,4%	29	59,2%	12	18,8%	38	74,5%	36	72,0%	56	70,0%	68	69,4%	27	50,0%	30	71,4%
**Fear**	**Very**	197	35,8%	21	36,8%	8	16,3%	53	82,8%	26	51,0%	15	30,0%	47	58,8%	8	8,2%	13	21,7%	6	14,3%
	**Problematic**	190	34,5%	4	7,0%	30	61,2%	11	17,2%	20	39,2%	14	28,0%	29	36,3%	28	28,6%	45	75,0%	9	21,4%
	**Not**	164	29,8%	32	56,1%	11	22,4%	0	0,0%	5	9,8%	21	42,0%	4	5,0%	62	63,3%	2	3,3%	27	64,3%
**Knowledge/ Awareness**	**Very**	124	22,5%	16	28,1%	31	63,3%	0	0,0%	8	15,7%	9	18,0%	8	10,0%	25	25,5%	0	0,0%	27	64,3%
	**Problematic**	181	32,8%	13	22,8%	16	32,7%	31	48,4%	29	56,9%	5	10,0%	27	33,8%	10	10,2%	43	71,7%	7	16,7%
	**Not**	246	44,6%	28	49,1%	2	4,1%	33	51,6%	14	27,5%	36	72,0%	45	56,3%	63	64,3%	17	28,3%	8	19,0%
**Trust in Health Workers**	**Very**	96	17,4%	16	28,1%	2	4,1%	5	7,8%	15	29,4%	8	16,0%	8	10,0%	25	25,5%	0	0,0%	17	40,5%
	**Problematic**	73	13,2%	4	7,0%	4	8,2%	19	29,7%	17	33,3%	3	6,0%	1	1,3%	5	5,1%	20	33,3%	0	0,0%
	**Not**	382	69,3%	37	64,9%	43	87,8%	40	62,5%	19	37,3%	39	78,0%	71	88,8%	68	69,4%	40	66,7%	25	59,5%

Supplement C1 Reported access to chemotherapy items per country

Full cohort, n=553

		All countries		Congo		Ethiopia		Gabon		Kenya		Mali (n=39)		Nigeria		Tanzania		Uganda		Zimbabwe	
		**n**	**%**	**n**	**%**	**n**	**%**	**n**	**%**	**n**	**%**	**n**	**%**	**n**	**%**	**n**	**%**	**n**	**%**	**n**	**%**
**Availability of Chemotherapy Facility**	**Very problematic**	34	8,5%	9	18,8%	4	13,8%	1	2,8%	3	7,7%	1	2,6%	10	15,4%	3	4,8%	2	3,8%	1	3,8%
	**Problematic**	27	6,8%	0	0,0%	2	6,9%	1	2,8%	3	7,7%	4	10,3%	7	10,8%	4	6,3%	6	11,3%	0	0,0%
	**Not Problematic**	337	84,7%	39	81,3%	23	79,3%	34	94,4%	33	84,6%	34	87,2%	48	73,8%	56	88,9%	45	84,9%	25	96,2%
**Cost of Transport**	**Very problematic**	52	13,0%	39	81,3%	0	0,0%	0	0,0%	3	7,7%	1	2,6%	5	7,7%	1	1,6%	1	1,9%	2	7,7%
	**Problematic**	137	34,3%	5	10,4%	23	79,3%	3	8,3%	23	59,0%	18	46,2%	30	46,2%	20	31,7%	13	24,1%	2	7,7%
	**Not Problematic**	210	52,6%	4	8,3%	6	20,7%	33	91,7%	13	33,3%	20	51,3%	30	46,2%	42	66,7%	40	74,1%	22	84,6%
**Availability of Transport**	**Very problematic**	19	4,8%	9	18,8%	2	6,9%	1	2,8%	0	0,0%	1	2,6%	4	6,2%	1	1,6%	0	0,0%	1	3,8%
	**Problematic**	90	22,6%	4	8,3%	19	65,5%	2	5,6%	11	28,2%	7	17,9%	20	30,8%	18	28,6%	6	11,3%	3	11,5%
	**Not Problematic**	289	72,6%	35	72,9%	8	27,6%	33	91,7%	28	71,8%	31	79,5%	41	63,1%	44	69,8%	47	88,7%	22	84,6%
**Cost of Absence from Home**	**Very problematic**	47	11,8%	33	68,8%	0	0,0%	0	0,0%	3	7,7%	1	2,6%	4	6,2%	4	6,3%	0	0,0%	2	7,7%
	**Problematic**	128	32,1%	11	22,9%	12	41,4%	6	16,7%	20	51,3%	11	28,2%	32	49,2%	20	31,7%	15	27,8%	1	3,8%
	**Not Problematic**	224	56,1%	4	8,3%	17	58,6%	30	83,3%	16	41,0%	27	69,2%	29	44,6%	39	61,9%	39	72,2%	23	88,5%
**Cost of Treatment**	**Very problematic**	118	29,6%	45	93,8%	10	34,5%	1	2,8%	12	30,8%	2	5,1%	37	56,9%	9	14,3%	1	1,9%	1	3,8%
	**Problematic**	144	36,1%	0	0,0%	14	48,3%	6	16,7%	26	66,7%	21	53,8%	18	27,7%	25	39,7%	18	33,3%	16	61,5%
	**Not Problematic**	137	34,3%	3	6,3%	5	17,2%	29	80,6%	1	2,6%	16	41,0%	10	15,4%	29	46,0%	35	64,8%	9	34,6%
**Leaving Home for Treatment**	**Very problematic**	41	10,3%	13	27,1%	4	13,8%	2	5,6%	2	5,1%	13	33,3%	4	6,2%	2	3,2%	0	0,0%	1	3,8%
	**Problematic**	102	25,6%	4	8,3%	7	24,1%	2	5,6%	7	17,9%	25	64,1%	26	40,0%	16	25,4%	14	25,9%	1	3,8%
	**Not Problematic**	256	64,2%	31	64,6%	18	62,1%	32	88,9%	30	76,9%	1	2,6%	35	53,8%	45	71,4%	40	74,1%	24	92,3%
**Waiting Time**	**Very problematic**	19	4,8%	7	14,6%	1	3,4%	0	0,0%	1	2,6%	1	2,6%	6	9,2%	2	3,2%	0	0,0%	1	3,8%
	**Problematic**	100	25,1%	5	10,4%	2	6,9%	3	8,3%	19	48,7%	14	35,9%	15	23,1%	11	17,5%	27	50,0%	4	15,4%
	**Not Problematic**	280	70,2%	36	75,0%	26	89,7%	33	91,7%	19	48,7%	24	61,5%	44	67,7%	50	79,4%	27	50,0%	21	80,8%
**Use of Alternative Medicine**	**Very problematic**	75	19.0%	20	41,7%	3	10,3%	24	66,7%	4	10,3%	3	7,7%	9	13,8%	7	11,1%	2	4,0%	3	11,5%
	**Problematic**	84	21.3%	8	16,7%	8	27,6%	5	13,9%	3	7,7%	11	28,2%	4	6,2%	15	23,8%	21	42,0%	9	34,6%
	**Not Problematic**	236	59.7%	20	41,7%	18	62,1%	7	19,4%	32	82,1%	25	64,1%	52	80,0%	41	65,1%	27	54,0%	14	53,8%
**Fear**	**Very problematic**	141	35,3%	18	37,5%	7	24,1%	29	80,6%	16	41,0%	14	35,9%	33	50,8%	8	12,7%	11	20,4%	5	19,2%
	**Problematic**	162	40,6%	2	4,2%	21	72,4%	7	19,4%	18	46,2%	13	33,3%	28	43,1%	23	36,5%	41	75,9%	9	34,6%
	**Not Problematic**	96	24,1%	28	58,3%	1	3,4%	0	0,0%	5	12,8%	12	30,8%	4	6,2%	32	50,8%	2	3,7%	12	46,2%
**Knowledge/ Awareness**	**Very problematic**	53	13,3%	13	27,1%	14	48,3%	0	0,0%	2	5,1%	2	5,1%	6	9,2%	4	6,3%	0	0,0%	12	46,2%
	**Problematic**	156	39,1%	13	27,1%	14	48,3%	20	55,6%	25	64,1%	5	12,8%	26	40,0%	7	11,1%	39	72,2%	7	26,9%
	**Not Problematic**	190	47,6%	22	45,8%	1	3,4%	16	44,4%	12	30,8%	32	82,1%	33	50,8%	52	82,5%	15	27,8%	7	26,9%
**Trust in Health Workers**	**Very problematic**	41	10,3%	13	27,1%	1	3,4%	3	8,3%	11	28,2%	2	5,1%	6	9,2%	3	4,8%	0	0,0%	2	7,7%
	**Problematic**	54	13,5%	4	8,3%	2	6,9%	13	36,1%	13	33,3%	1	2,6%	1	1,5%	3	4,8%	17	31,5%	0	0,0%
	**Not Problematic**	304	76,2%	31	64,6%	26	89,7%	20	55,6%	15	38,5%	36	92,3%	58	89,2%	57	90,5%	37	68,5%	24	92,3%

Supplement C2 Reported access to Chemotherapy items per country

Sub-cohort “chemotherapy recommended”, n = 399

		All countries		Breast (n=116)		Cervix (n=118)		Colorectal (n=89)		NHL (n=73)		Prostate (n=107)		Kaposi (n=49)	
		n	%	n	%	n	%	n	%	n	%	n	%	n	%
Availability of Chemotherapy Facility	Very problematic	83	15,1%	18	15,7%	19	16,1%	9	10,1%	11	15,3%	24	22,4%	2	4,1%
	Problematic	35	6,4%	10	8,7%	9	7,6%	5	5,6%	3	4,2%	5	4,7%	3	6,1%
	Not Problematic	432	78,5%	87	75,7%	90	76,3%	75	84,3%	58	80,6%	78	72,9%	44	89,8%
Cost of Transport	Very problematic	111	20,1%	22	19,1%	21	17,8%	14	15,7%	18	24,7%	33	30,8%	3	6,1%
	Problematic	166	30,1%	42	36,5%	39	33,1%	22	24,7%	29	39,7%	22	20,6%	12	24,5%
	Not Problematic	274	49,7%	51	44,3%	58	49,2%	53	59,6%	26	35,6%	52	48,6%	34	69,4%
Availability of Transport	Very problematic	64	11,6%	12	10,4%	11	9,3%	7	7,9%	10	13,7%	22	20,6%	2	4,1%
	Problematic	113	20,5%	32	27,8%	27	22,9%	12	13,5%	18	24,7%	18	16,8%	6	12,2%
	Not Problematic	374	67,9%	71	61,7%	80	67,8%	70	78,7%	45	61,6%	67	62,6%	41	83,7%
Cost of Absence from Home	Very problematic	107	19,4%	24	20,9%	15	12,7%	13	14,6%	18	24,7%	34	31,8%	3	6,1%
	Problematic	149	27,0%	33	28,7%	41	34,7%	25	28,1%	23	31,5%	13	12,1%	14	28,6%
	Not Problematic	295	53,5%	58	50,4%	62	52,5%	51	57,3%	32	43,8%	60	56,1%	32	65,3%
Cost of Treatment	Very problematic	185	33,6%	38	33,0%	46	39,0%	26	29,2%	29	39,7%	42	39,3%	4	8,2%
	Problematic	177	32,1%	44	38,3%	38	32,2%	34	38,2%	18	24,7%	20	18,7%	23	46,9%
	Not Problematic	189	34,3%	33	28,7%	34	28,8%	29	32,6%	26	35,6%	45	42,1%	22	44,9%
Leaving Home for Treatment	Very problematic	90	16,3%	19	16,5%	15	12,7%	18	20,2%	13	17,8%	23	21,5%	2	4,1%
	Problematic	132	24,0%	37	32,2%	33	28,0%	11	12,4%	17	23,3%	22	20,6%	12	24,5%
	Not Problematic	329	59,7%	59	51,3%	70	59,3%	60	67,4%	43	58,9%	62	57,9%	35	71,4%
Waiting Time	Very problematic	83	15,1%	15	13,0%	16	13,6%	12	13,5%	8	11,0%	30	28,0%	2	4,1%
	Problematic	117	21,2%	23	20,0%	30	25,4%	19	21,3%	16	21,9%	20	18,7%	9	18,4%
	Not Problematic	351	63,7%	77	67,0%	72	61,0%	58	65,2%	49	67,1%	57	53,3%	38	77,6%
Use of Alternative Medicine	Very problematic	118	21,7%	18	15,7%	32	27,4%	19	21,3%	12	16,4%	27	26,5%	10	20,4%
	Problematic	108	19,8%	21	18,3%	26	22,2%	14	15,7%	19	26,0%	19	18,6%	9	18,4%
	Not Problematic	319	58,5%	76	66,1%	59	50,4%	56	62,9%	42	57,5%	56	54,9%	30	61,2%
Fear	Very problematic	197	35,8%	35	30,4%	49	41,5%	29	32,6%	27	37,0%	42	39,3%	15	30,6%
	Problematic	190	34,5%	49	42,6%	42	35,6%	38	42,7%	19	26,0%	23	21,5%	19	38,8%
	Not Problematic	164	29,8%	31	27,0%	27	22,9%	22	24,7%	27	37,0%	42	39,3%	15	30,6%
Knowledge/ Awareness	Very problematic	124	22,5%	28	24,3%	27	22,9%	9	10,1%	18	24,7%	35	32,7%	7	14,3%
	Problematic	181	32,8%	35	30,4%	33	28,0%	41	46,1%	26	35,6%	26	24,3%	20	40,8%
	Not Problematic	246	44,6%	52	45,2%	58	49,2%	39	43,8%	29	39,7%	46	43,0%	22	44,9%
Trust in Health Workers	Very problematic	96	17,4%	21	18,3%	16	13,6%	12	13,5%	11	15,1%	32	29,9%	4	8,2%
	Problematic	73	13,2%	14	12,2%	16	13,6%	12	13,5%	9	12,3%	14	13,1%	8	16,3%
	Not Problematic	382	69,3%	80	69,6%	86	72,9%	65	73,0%	53	72,6%	61	57,0%	37	75,5%

Supplement D1 Reported access to Chemotherapy items per cancer entity

Full cohort, n=553; Kaposi, Kaposi's sarcoma; NHL, non-Hodgkin lymphoma

		All countries		Breast		Cervix (n=118)		Colorectal		NHL (n=73)		Prostate		Kaposi	
		n	%	n	%	n	%	n	%	n	%	n	%	n	%
**Availability of Chemotherapy Facility**	**Very**	83	15,1%	18	15,7%	19	16,1%	9	10,1%	11	15,3%	24	22,4%	2	4,1%
	**Problematic**	35	6,4%	10	8,7%	9	7,6%	5	5,6%	3	4,2%	5	4,7%	3	6,1%
	**Not**	432	78,5%	87	75,7%	90	76,3%	75	84,3%	58	80,6%	78	72,9%	44	89,8%
**Cost of Transport**	**Very**	111	20,1%	22	19,1%	21	17,8%	14	15,7%	18	24,7%	33	30,8%	3	6,1%
	**Problematic**	166	30,1%	42	36,5%	39	33,1%	22	24,7%	29	39,7%	22	20,6%	12	24,5%
	**Not**	274	49,7%	51	44,3%	58	49,2%	53	59,6%	26	35,6%	52	48,6%	34	69,4%
**Availability of Transport**	**Very**	64	11,6%	12	10,4%	11	9,3%	7	7,9%	10	13,7%	22	20,6%	2	4,1%
	**Problematic**	113	20,5%	32	27,8%	27	22,9%	12	13,5%	18	24,7%	18	16,8%	6	12,2%
	**Not**	374	67,9%	71	61,7%	80	67,8%	70	78,7%	45	61,6%	67	62,6%	41	83,7%
**Cost of Absence from Home**	**Very**	107	19,4%	24	20,9%	15	12,7%	13	14,6%	18	24,7%	34	31,8%	3	6,1%
	**Problematic**	149	27,0%	33	28,7%	41	34,7%	25	28,1%	23	31,5%	13	12,1%	14	28,6%
	**Not**	295	53,5%	58	50,4%	62	52,5%	51	57,3%	32	43,8%	60	56,1%	32	65,3%
**Cost of Treatment**	**Very**	185	33,6%	38	33,0%	46	39,0%	26	29,2%	29	39,7%	42	39,3%	4	8,2%
	**Problematic**	177	32,1%	44	38,3%	38	32,2%	34	38,2%	18	24,7%	20	18,7%	23	46,9%
	**Not**	189	34,3%	33	28,7%	34	28,8%	29	32,6%	26	35,6%	45	42,1%	22	44,9%
**Leaving Home for Treatment**	**Very**	90	16,3%	19	16,5%	15	12,7%	18	20,2%	13	17,8%	23	21,5%	2	4,1%
	**Problematic**	132	24,0%	37	32,2%	33	28,0%	11	12,4%	17	23,3%	22	20,6%	12	24,5%
	**Not**	329	59,7%	59	51,3%	70	59,3%	60	67,4%	43	58,9%	62	57,9%	35	71,4%
**Waiting Time**	**Very**	83	15,1%	15	13,0%	16	13,6%	12	13,5%	8	11,0%	30	28,0%	2	4,1%
	**Problematic**	117	21,2%	23	20,0%	30	25,4%	19	21,3%	16	21,9%	20	18,7%	9	18,4%
	**Not**	351	63,7%	77	67,0%	72	61,0%	58	65,2%	49	67,1%	57	53,3%	38	77,6%
**Use of Alternative Medicine**	**Very**	118	21,7%	18	15,7%	32	27,4%	19	21,3%	12	16,4%	27	26,5%	10	20,4%
	**Problematic**	108	19,8%	21	18,3%	26	22,2%	14	15,7%	19	26,0%	19	18,6%	9	18,4%
	**Not**	319	58,5%	76	66,1%	59	50,4%	56	62,9%	42	57,5%	56	54,9%	30	61,2%
**Fear**	**Very**	197	35,8%	35	30,4%	49	41,5%	29	32,6%	27	37,0%	42	39,3%	15	30,6%
	**Problematic**	190	34,5%	49	42,6%	42	35,6%	38	42,7%	19	26,0%	23	21,5%	19	38,8%
	**Not**	164	29,8%	31	27,0%	27	22,9%	22	24,7%	27	37,0%	42	39,3%	15	30,6%
**Knowledge/ Awareness**	**Very**	124	22,5%	28	24,3%	27	22,9%	9	10,1%	18	24,7%	35	32,7%	7	14,3%
	**Problematic**	181	32,8%	35	30,4%	33	28,0%	41	46,1%	26	35,6%	26	24,3%	20	40,8%
	**Not**	246	44,6%	52	45,2%	58	49,2%	39	43,8%	29	39,7%	46	43,0%	22	44,9%
**Trust in Health Workers**	**Very**	96	17,4%	21	18,3%	16	13,6%	12	13,5%	11	15,1%	32	29,9%	4	8,2%
	**Problematic**	73	13,2%	14	12,2%	16	13,6%	12	13,5%	9	12,3%	14	13,1%	8	16,3%
	**Not**	382	69,3%	80	69,6%	86	72,9%	65	73,0%	53	72,6%	61	57,0%	37	75,5%

Supplement D2 Reported access to Chemotherapy items per cancer entity

Sub-cohort "chemotherapy recommended", n= 399, Kaposi, Kaposi's sarcoma; NHL, non-Hodgkin lymphoma

**Introducing question:** Did any of the following reasons hinder, delay or allow to receive chemotherapy? (To ask ALL patients)				
**Dimension**	**Item name**	**Item definition**	**Options**	**Comment**
Availability	Availability of Chemotherapy Facility	Health facilities that can offer chemotherapy in the area	0. Not available<br>1. Problematic<br>2. Available	
Accessibility	Cost of Transport	Costs for transportation to chemotherapy treatment	0. Not affordable<br>1. Problematic<br>2. Affordable	
	Availability of Transport	Public transportation system/private transport to reach the chemotherapy treatment	0. Not accessible<br>1. Problematic<br>2. Accessible	
Affordability	Cost of Treatment	Costs for everything concerning chemotherapy in the hospital	0. Not affordable<br>1. Problematic<br>2. Affordable	
	Cost of Absence from Home	Costs of being absent from home (e.g. because of work, costs for childcare, etc.)	0. Not affordable<br>1. Problematic<br>2. Affordable	
Accommodation	Waiting Time	Obtaining appointment with reasonable waiting time	0. Not possible (no appointment)<br>1. Problematic (appointment was given but long waiting time)<br>2. Possible (appointment with reasonable waiting time)	
	Leaving Home for Treatment	Leaving the family home for chemotherapy	0. Not possible<br>1. Problematic<br>2. Possible	
Affordability	Use of Alternative Medicine	Use of traditional/alternative medicine as a replacement of other medication	0. Not present<br>1. Problematic (alternative medicine sometimes used as replacement)<br>2. Present (continuously used instead of other medication)	OPPOSITE ORDINAL SCALE: CHANGE SEQUENCE FOR ANALYSIS
	Fear	Fear about complications, side effects and/or consequences of the chemotherapy	0. Not present<br>1. Problematic (slightly afraid)<br>2. Present (fear)	OPPOSITE ORDINAL SCALE: CHANGE SEQUENCE FOR ANALYSIS
	Knowledge/ Awareness	Feeling of awareness and knowledge about the cancer and the chemotherapy treatment	0. Not present<br>1. Problematic (partly informed)<br>2. Present	
	Trust in Health Workers	Believe/sympathy/trust in the health workers	0. Not present<br>1. Problematic<br>2. Present	

Supplement E: Questionnaire items - Access to Chemotherapy

Patients were initially asked the introducing question and then the Item definitions plus opt

Variable		Availability<br>Availability of CT Facility	Accessibility<br>Cost of Transport	 Availability of Transport	Affordability<br>Cost of Absence from Home	 Cost of Treatment	Accomodation<br>Leaving Home for Treatment	 Waiting Time	Acceptability<br>Use of Alternative Medicine	 Fear	 Lack of Information	 Trust in Health Workers
		OR (95%-CI)	OR 95%-CI	OR 95%-CI	OR 95%-CI	OR 95%-CI	**OR 95%-CI**	OR 95%-CI	OR 95%-CI	OR 95%-CI	OR 95%-CI	OR 95%-CI
**Education & Wealth, n=399**	Higher score (0 to 6)	1.0<br>3 (0.79, 1.35)	**0.63 (0.5, 0.78)**	**0.6<br>6 (0.51, 0.85)**	**0.78 (0.63, 0.95)**	**0.7<br>1 (0.58, 0.86)**	**(0.64,<br>0.8 0.98)**	0.9<br>3 (0.75, 1.14)	0.7<br>9 (0.63, 0.98)	**1.22 (1.02, 1.46)**	1.0<br>9 (0.91, 1.31)	1.0<br>5 (0.82, 1.33)
**HDI, n=399**	Higher HDI (steps of 0.1)	0.7<br>6 (0.49, 1.17)	0.79 (0.59, 1.06)	**0.6<br>9 (0.48, 0.98)**	1.03 (0.77, 1.38)	0.8<br>2 (0.62, 1.09)	**(0.15,<br>0.23 0.35)**	**0.7<br>2 (0.51, 1.0)**	**2.5<br>2 (1.83, 3.47)**	**1.65 (1.24, 2.21)**	1.2<br>4 (0.94, 1.64)	**2.3<br>1 (1.64, 3.25)**
**Responding person (self*, n=184)**	Other, n=215	**2.9<br>7 (1.54, 5.73)**	**1.86 (1.22, 2.85)**	1.2<br>9 (0.79, 2.12)	**1.92 (1.26, 2.93)**	**1.7<br>9 (1.19, 2.68)**	**(1.17,<br>1.86 2.97)**	1.4 (0.88, 2.24)	1.1 (0.7, 1.71)	1.38 (0.92, 2.07)	1.1<br>9 (0.79, 1.8)	1.5<br>3 (0.91, 2.58)
**Sex (female*, n=265)**	Male, n=134	0.6<br>3 (0.25, 1.61)	1.28 (0.69, 2.37)	1.1<br>5 (0.56, 2.37)	1.45 (0.79, 2.67)	1.5<br>8 (0.9, 2.77)	(0.57,<br>1.14 2.27)	1.3<br>1 (0.67, 2.59)	0.8 (0.43, 1.49)	**0.45 (0.25, 0.8)**	0.6<br>1 (0.34, 1.07)	1.5<br>5 (0.73, 3.26)
**Age, n=399**	Higher age	1.0 (0.98, 1.02)	0.98 (0.97, 1.0)	0.9<br>9 (0.97, 1.0)	0.99 (0.98, 1.01)	0.9<br>9 (0.98, 1.01)	(0.98,<br>0.99 1.01)	0.9<br>9 (0.97, 1.01)	1.0<br>1 (0.99, 1.02)	0.99 (0.98, 1.01)	1.0 (0.99, 1.02)	1.0 (0.98, 1.02)
**Entity (*NHL, n =61)**												
**Breast (n=100)**		1.4<br>5 (0.55, 3.82)	0.93 (0.46, 1.87)	1.1 (0.5, 2.43)	1.1 (0.54, 2,24)	1.6<br>5 (0.84, 3.25)	(0.84,<br>1.84 4.03)	1.3<br>6 (0.61, 3.03)	0.5<br>9 (0.29, 1.22)	0.99 (0.5, 1.94)	0.8<br>1 (0,41, 1.59)	1.6<br>7 (0.68, 4.13)
**Cervix (n=84)**		1.1<br>9 (0.44, 3.17)	0.73 (0.36, 1.49)	0.6<br>6 (0.29, 1.51)	0.92 (0.45, 1.87)	1.8<br>8 (0.94, 3.75)	1.13 (0.5, 2.52)	1.2<br>9 (0.57, 2.88)	1.1<br>5 (0.56, 2.35)	1.4 (0,7, 2.8)	0.5<br>9 (0.3, 1.18)	1.0<br>4 (0.4, 2.71)
**Colorectum (n=77)**		0.9<br>9 (0.37, 2.7)	0.61 (0.31, 1.21)	0.5<br>9 (0.27, 1.31)	0.77 (0.39, 1.54)	1.3<br>5 (0.7, 2.6)	(0.52,<br>1.13 2.45)	1.3<br>6 (0.64, 2.87)	0.8 (0.4, 1.61)	1.23 (0.64, 2.37)	0.8<br>1 (0.42, 1.54)	1.4 (0.61, 3.24)
**Kaposi Sarcoma (n=37)**		0.4<br>1 (0.08, 2.11)	**0.27 (0.11, 0,66)**	0.3<br>8 (0.13, 1.08)	0.46 (0.19, 1.06)	0.6<br>2 (0.29, 1.36)	(0.42,<br>1.07 2.78)	0.5<br>9 (0.22, 1.64)	0.4<br>1 (0.17, 0.99)	0.61 (0.28, 1.35)	1.1<br>5 (0.52, 2.52)	0.7<br>4 (0.26, 2.13)
**Prostate (n=40)**		0.6<br>8 (0.14, 3.22)	0.98 (0.39, 2.46)	0.9<br>5 (0.34, 2.68)	0.68 (0.26, 1.74)	0.7<br>1 (0.29, 1.75)	(0.53,<br>1.44 3.93)	1.7<br>9 (0.68, 4.71)	0.8 (0.31, 2.05)	**3.48 (1.42, 8.5)**	0.7 (0,29, 1.67)	2.4<br>9 (0.89, 6.94)

Supplement F: Associations with barrier report of participants who were recommended chemotherapy

*Sensitivity analysis in a subset of patients who either received chemotherapy or were recommended chemotherapy but did not receive it (n=399): ORs (adjusted for all items mentioned) for factors influencing report of barriers in access of chemotherapy. ORs > 1 indicate increased problems. *, reference category; CI, confidence interval; HDI, human development index of the country; NHL, non-Hodgkin lymphoma; OR, odds ratio; CT, chemotherapy.*
